# Utility of CD44/CD24 in the Outcome and Prognosis of Oral Squamous Cell Carcinoma: A Systematic Review

**DOI:** 10.7759/cureus.42899

**Published:** 2023-08-03

**Authors:** Reshma Poothakulath Krishnan, Deepak Pandiar, Pratibha Ramani, Karthikeyan Ramalingam, Selvaraj Jayaraman

**Affiliations:** 1 Oral Pathology and Microbiology, Saveetha Dental College and Hospitals, Saveetha Institute of Medical and Technical Sciences, Saveetha University, Chennai, IND; 2 Centre of Molecular Medicine and Diagnostics (COMManD) Department of Biochemistry, Saveetha Dental College and Hospitals, Saveetha Institute of Medical and Technical Sciences, Saveetha University, Chennai, IND

**Keywords:** survival, prognosis, oral squamous cell carcinoma, cd44, cd24

## Abstract

Cancer stem cells (CSCs) are characterized by their capacity for self-renewal and differentiation. CD44 and CD24 are two commonly used markers to identify these CSCs. Despite the enormous amount of data available in the literature, their specificity and coexistence remain elusive in oral squamous cell carcinoma (OSCC). In the present review, we aimed to assess the diagnostic utility of the CD44/CD24 combination in tumor development and metastasis in OSCC. Two investigators independently performed a systematic search to identify all the relevant studies from various electronic databases. Out of 694 articles, 9 were found eligible for further evaluation. Details including the number of patients, gender, site, tobacco and alcohol consumption, histological stage, CD24 expression, CD44 expression, CD44/CD24 expression, nodal status, disease-free survival, and overall survival were extracted. CD44+CD24- expression was noted in 35/207 (16.9%) cases, CD44+CD24+ in 53/207 (25.6%) cases, CD44-CD24- in 49/207 subjects (23.67%), and CD44-/CD24+ in 70/207 (33.81%) cases. CD44 or CD24 or their co-expression did not correlate with the disease-free survival rate, and double negatives (CD44-/CD24-) demonstrated a higher overall survival than other immunotypes. CD44/CD24 profile may be used on small incisional biopsies to predict the outcome and treatment planning. This finding may help in developing new therapeutic targets to suppress cancer metastasis and provide a better long-term prognosis for patients diagnosed with OSCC.

## Introduction and background

Oral squamous cell carcinoma (OSCC) accounts for about 95% of oral malignant lesions in developing countries [1]. Second primary tumors and loco-regional recurrence have an impact on these patients' long-term prognosis, and unfortunately, the mortality rates have remained stable in recent years (approximately 50% for the past 40 years) [2]. As a result, there is considerable interest in identifying various prognostic factors to guide clinicians in treating OSCCs. An emerging concept of carcinogenesis contends that cancer stem cells (CSCs) are important in determining the biological characteristics of cancer, like metastasis, growth, and invasion [3]. These groups of cells show capacity for self-renewal, tumorigenicity, differentiation, and exhibit properties of both stem cells and tumor cells [4]. Expression of cell surface markers such as CD29, CD44, CD90, ESA, CD24, ALDH1, and CD133 is used to identify the CSCs [5]. These cells also contribute to chemoresistance and radioresistance, further increasing the chance of metastatic spread and locoregional recurrence.

CD44, a transmembrane cell surface receptor, plays an important role in the interaction between the extracellular matrix (ECM) and malignant cells [6]. The constituents of the ECM, like metalloproteinases, hyaluronan, osteopontin, and collagens, bind to this glycoprotein and activate various pathways. Among these, hyaluronan, which contributes to cell adhesion and migration, is considered the immediate ligand for CD44 [7]. Increased CD44 expression is associated with poor prognosis, cancer progression, and metastasis [8]. This protein is widely used in identifying CSCs in various tumors like breast carcinomas, colorectal cancers, and bronchoalveolar carcinomas including OSCC [7]. CD24 is another important CSC marker. It is a heavily glycosylated surface protein anchored by glycosyl-phosphatidyl inositol [9]. CD24 is over-expressed in various tumors like non-small cell carcinoma, colorectal, breast, renal, pancreatic, bladder cancers, and OSCC [10]. This protein is important in cancer adhesion, proliferation, and metastasis by facilitating interactions with endothelial cells. CD44 and CD24 gained considerable interest in oncology, and a combination of these two markers is believed to characterize various tumors, including OSCC [11]. They are engaged in specific functions during tumor progression and metastasis. Despite extensive studies on CD44 and CD24, their coexistence, correlation, and specificity still remain elusive. In the present review, we aimed to assess the diagnostic utility of the CD44/CD24 combination in tumor development and metastasis in OSCC.

## Review

Materials and methods

This systematic review of CD44/CD24 in OSCC was carried out according to the PRISMA (Preferred Reporting Items for Systematic Reviews and Meta-Analyses) guidelines. The study was registered in PROSPERO (CRD42023397220). Based on Population, Intervention, Comparison, Outcome, and Study (PICOS), the review question was “Does CD44/CD24 expression determine the prognostic outcome in patients with oral squamous cell carcinoma?”

Inclusion and Exclusion Criteria

Original research articles published in English that evaluated the expression of CD44/CD24 in OSCC regardless of age, gender, socioeconomic status, and ethnicity were included in this study. Systematic reviews, reviews, animal studies, research articles that included cell lines, and articles where full length was not obtained, were excluded. Studies with missing data, duplicates using the same data, and patients with oral cavity metastatic lesions were also not included. 

Search Strategy and Data Bases

Two investigators (RPK and DP) independently performed a systematic search to identify all the relevant studies in PubMed, Scopus, Google Scholar, EMBASE, Web of Science, and Cochrane databases as of February 25, 2023, without any period restriction. The following keywords were used: "CD24," "CD44," "oral squamous cell carcinoma," and "OSCC." The following search strategy was constructed: ((((((oral squamous cell carcinoma[Title/Abstract]) OR (oral squamous cell carcinoma[MeSH Terms])) OR (OSCC[Title/Abstract])) OR (OSCC[MeSH Terms])) OR (OSCC[MeSH Terms])) AND ((CD24[Title/Abstract]) OR (CD24[MeSH Terms]))) AND ((CD44[Title/Abstract]) OR (CD44[MeSH Terms])). A manual search was conducted that included the reference lists from the relevant articles. All the article titles and abstracts were initially screened, and those that did not meet the inclusion criteria were filtered out. Later, investigators retrieved and reviewed the full texts of all potentially eligible articles along with the supplementary data. Any disagreements regarding the inclusion of articles were discussed and resolved by uniform consensus, and the list of articles to be included in this systematic review was finalized.

Data Extraction

RPK and DP independently reviewed the full texts of all the included articles, and the following data were extracted: the number of patients, gender, primary site, tobacco consumption, alcohol consumption, histological stage, CD24 expression, CD44 expression, CD44/CD24 expression, nodal status, disease-free survival, and overall survival.

Results

Search Results

**Figure 1:**
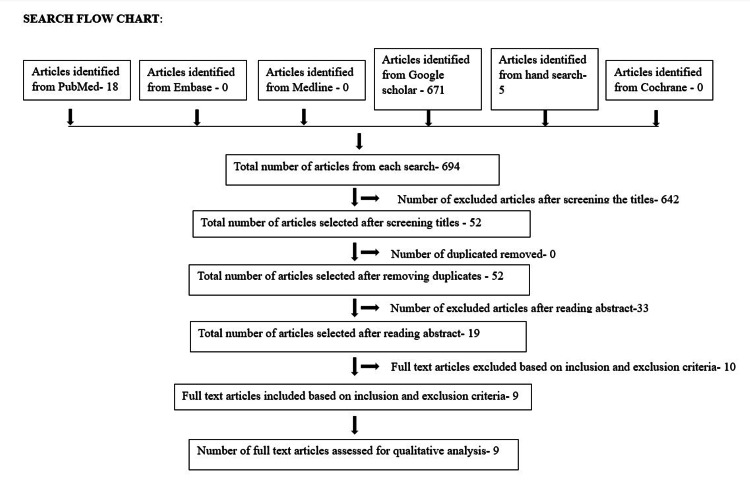
PRISMA flow chart (search flow chart) PRISMA, Preferred Reporting Items for Systematic Reviews and Meta-Analyses

We screened 694 articles (18 from PubMed, 671 from Google Scholar, and five from hand search). A total of 642 articles were removed based on the initial assessment of titles, and 33 articles were removed after reading the abstracts. Ten articles were further removed by RPK and DP after reading the entire manuscript. Out of 694 articles, nine were found eligible for further evaluation and systematic analysis (Figure 1).

Clinicopathological Data

All the articles included in this research were observational, cross-sectional studies. Details of the included studies are mentioned in Table 1, Table 2, and Table 3 [11-19]. A total of 555 patients with OSCC were included in the present systematic review. Six articles reported the gender distribution of the study subjects, males (72.29%; 214) outnumbered females (27.7%; 82) with a ratio of 2.6:1 (Table 1). Most of the cases were from the tongue (42.70%), floor of mouth (16.78%), and gingiva (9.48%). Oliveira LR et al., Saleem S et al., and Han J et al. mentioned the history of tobacco and alcohol consumption of the included patients [13,16,17]. Out of 166 patients, 90.96% had a history of tobacco use and 86.74% had a history of alcohol consumption (Table 1).

**Table 1:** Demographic details of the included studies SCC, squamous cell carcinoma; OSCC, oral squamous cell carcinoma

		No. of SCC patients	Gender		Site											Tobacco		Alcohol	
			Male	Female	Tongue	Oral floor	Upper gingiva	Lower gingiva	Buccal mucosa	Hard palate	Lower lip	Base of tongue	Floor of mouth	Retromolar trigone	Others	Yes	No	Yes	No
1	Mirhashemi M et al. [11]	45	Not specified		Not specified											Not specified		Not specified	
2	Tamatani T et al. [12]	70	34	36	38	5	5	16	6							Not specified		Not specified	
3	Oliveira LR et al. [13]	157	136	21	54	41				18	14				30	146	11	140	17
4	Al-Magsoosi MJN et al. [14]	10	5	5	Not specified											Not specified		Not specified	
5	Abdulmajeed AA et al. [15]	176	Not specified		Not specified											Not specified		Not specified	
6	Saleem S et al. [16]	5	3	2	5											1	4	0	5
7	Han J et al. [17]	6	3	1					2			4				4	0	4	0
8	Todoroki K et al. [18]	50	33	17	Not specified											Not specified		Not specified	
9	de Moraes et al. [19]	36	Not specified for OSCC alone		20		5		1	4			4	2		Not specified specifically for OSCC		Not specified specifically for OSCC	

CD24 Expression in Oral Squamous Cell Carcinoma

CD24 expression data were clearly mentioned in 322 cases, with 197 (61.8%) showing positive immunohistochemical positivity. However, no difference in CD24 expression was noted between well-differentiated and moderately to poorly differentiated squamous cell carcinomas (PDSCC). CD24 immunopositivity was noted in 43.93% (29/69) of well-differentiated squamous cell carcinoma (WDSCC) and 44.89% (22/49) of moderately differentiated squamous cell carcinoma (MDSCC) + PDSCC (Table 2).

**Table 2:** Histopathological, CD44/CD24 expression, and prognostic data of the included studies OSCC, Oral squamous cell carcinoma; SCC, squamous cell carcinoma; PCR, polymerase chain reaction, QRT PCR, real-time quantitative reverse transcription polymerase chain reaction; WDSCC, well-differentiated squamous cell carcinoma; MDSCC, moderately differentiated squamous cell carcinoma; PDSCC, poorly differentiated squamous cell carcinoma; SMA, smooth muscle actin

		Method used	Sample size	Number of samples and different grades of OSCC	CD 24 expression in OSCC	CD 24-Findings	CD44 expression in OSCC	CD44- Findings	CD24/44 expression	Findings	Disease-free survival	Five-year overall survival
1	Mirhashemi M et al. [11]	Real-time quantitative reverse transcription PCR reaction	45	Low grade (Grade I, II): 20, high grade (Grade III): 25	Low-grade SCC - 7(35%) - low expression and 13 (65%) - high expression High-grade SCC - 5(20%) - low expression and 20 (80%) - high expression	33 (73%) OSCC show high expression	Low-grade SCC - 7(35%) - low expression and 13(65%) - high expression High-grade SCC-11 (44%) - low expression and 14(56%) - high expression	27 (60%) OSCC show high expression	Low-grade SCC-correlation coefficient-0.713, P<0.001 High-grade SCC-correlation coefficient-0.682, P<0.001	Significant correlation was evident in both the groups	Not mentioned	Not mentioned
2	Tamatani T et al. [12]	Immunohistochemistry	70	WDSCC - 35, MDSCC - 30, PDSCC - 3. YK1 - 5, YK2 - 24, YK3 - 30, YK4C - 6, YK4D - 3 (Yamamoto-Kohama classification)	41/70 (59%) positive for OSCC cases CD24 positive cases WDCC - 24, MDSCC - 15, PDSCC - 1 YK1 - 5, YK2 - 17, YK3 - 12, YK4C - 3, YK4D - 1	Not associated with tumor size, histological differentiation, or lymph node metastasis, disease-free survival state. Associated with YK classification (p=0.03). As the grade increases CD24 expression increases.	70/70(100%) CD44 and 70/70(100%) CD 4V9	All cases positive for CD44. CD44 and CD44v were localized to the plasma membrane and cytoplasm of cancer cells	No correlation done	No correlation done	CD24 did not show any association with disease-free survival rate (p=0.87)	Not mentioned
3	Oliveira LR et al. [13]	Immunohistochemistry	157	WDCC - 79, MDCC - 64, PDSCC - 14	112 cases positive for CD24	No association with prognosis. CD24 membrane localization was predominantly observed in well-differentiated areas.	CD44+ - 65 cases	CD44+ demonstrated a significant difference between the OS curves and was an independent factor of poor prognosis	CD44+: 17 (10.8%), CD24+: 64 (40.8%), CD44+/CD24+: 48 (30.6%), CD44-/CD24-: 28 (17.8%)	Double negative tumors demonstrate a higher OS than other immunophenotypes. No association with DFS. Five years. Overall survival was more in CD44-/24- (58.7%) followed by CD24+ (24.7%), CD44+ (10.8%) and CD44+/24+ (0%)	CD44+ (p-value-0.054), CD24+ 0.202, CD44+/CD24+: 0.311, CD44-/CD24-: 0.152 (no significant correlation in any immunophenotype)	CD44+ (10.8%), CD24+ (24.7%), CD44+/CD24+(0%), CD44-/CD24-(58.7%)
4	Al-Magsoosi MJN et al. [14]	Only immunohistochemical results included	10	10 - MDSCC		Expression of CD24 throughout the tumor epithelium and islands		Expression of CD44 throughout the tumor epithelium and islands	Expression of CD24 and CD44 correlated with each other p<0.005. It correlated with alpha SMA		Not mentioned	Not mentioned
5	Abdulmajeed AA et al. [15]	Immunohistochemistry	176	49+127 (pilot+independent validation)	Exact percentage/number is not clear	Training set - CD24 expression is more in OSCC than in normal and dysplastic tissue groups (p<0.008). CD24 expression increased with disease severity. Independent validation sample: CD 24 was effective in distinguishing OSCC from non-malignant tissues	CD44 positive in 49 piolet study samples	Training set-CD44 showed higher median stain intensity scores for OSCC than normal tissue (p<0.008). Irregular and disorganized staining in all cases	No correlation done	No correlation done	Not mentioned	Not mentioned
6	Saleem S et al. [16]	Cell line and Immunohistochemistry. Only immunohistochemistry was considered for further analysis	5	WDSCC - 1, MDSCC - 3, 1 - Not detected	Exact percentage/number is not clear	The cells in the basal layer expressing CD44 were negative for CD24	Exact percentage/number is not clear	CD44 expression increased from hyperplastic tissue to neck node negative and neck node positive OSCC	No correlation done	No correlation done	Not mentioned clearly	Not mentioned clearly
7	Han J et al. [17]	QRT PCR and immunohistochemistry	6	6	Exact percentage/number is not clear		Exact percentage and number was not mentioned clearly				Not mentioned	Not mentioned
8	Todoroki K et al. [18]	Cell line and Immunohistochemistry. Only immunohistochemistry was considered for further analysis	50	WDSCC - 34, MDSCC+PDSCC - 16	WDSCC - 5/34, MDSCC+PDSCC - 6/16	Significant difference between the invasive and the non-invasive front-p<0.01. CD24 expression was significantly correlated with gender (p=0.01)	CD44v3 was considered, CD44 positive - 17/34, MDSCC+PDSCC - 6/16	Cd44v3+cases show poor OS compared to CD44v3- cases	WDSCC-CD44v3+CD24-: 14/34, CD44v3+CD24+: 3/34, CD44v3-CD24-: 15/34, CD44v3-/CD24+: 2/34, MDSCC+PDSCC-CD44v3+CD24: 4/16, CD44v3+CD24+: 2/16, CD44v3-CD24-: 6/16, CD44v3-/CD24+: 4/16	CD44v3+/CD24- cases showed significantly worse OS than CDv3+/CD24- cases	Not mentioned	CD44v3+/CD24- (poor survival)< CD44v3+/CD24+
9	de Moraes et al. [19]	Immunohistochemistry	36	Cannot be evaluated as not specifically mentioned for OSCC							Not mentioned specifically for OSCC	Not mentioned specifically for OSCC

CD44 Expression in Oral Squamous Cell Carcinoma

Only 321 of the 555 cases mentioned CD44 expression, with 211 (65.73%) showing CD44 immunopositivity. The data for CD44v (an alternative splice variant of CD44) was mentioned in 120 cases, out of which 93 (77.5%) showed positive immunoexpression. 52/69 (75.36%) cases of WDSCC and 9/19 (47.36%) of MDSCC + PDSCC showed either CD44 or CD44v positivity. Interestingly, increased CD44 expression was associated with well-differentiated carcinomas (Table 2).

CD44/CD24 Co-expression in Oral Squamous Cell Carcinoma

Only two articles, Oliveira LR et al. and Todoroki K et al. explicitly mentioned CD44 and CD24 immunohistochemical co-expression data [13,18]. CD44+CD24- expression was noted in 35/207 (16.9%) cases, CD44+CD24+ in 53/207 (25.6%), CD44-CD24- in 49/207 subjects (23.67%), and CD44-/CD24+ in 70/207 (33.81%) cases. In our review, predominantly, CD44-/CD24+ immunoexpression was reported in OSCC, followed by CD44+CD24+ and CD44-CD24- (Table 2).

CD44/CD24 Expression and Its Relation to Nodal Metastasis (N+)

Nodal metastasis is common in patients with OSCC and is an important prognostic factor. Out of nine, two articles evaluated the relationship between CD44/CD24 expression and nodal metastasis [12,18]. CD44+N+ was seen in 12%, CD44+N- in 34%, CD44-N+ in 2%, and CD44-N- in 52% cases. CD 24+N+ was noted in 9.16% (11), CD24+N- in 34.16% (41), CD24-N+ in 8.33% (10), and CD24-N- in 48.33% (58) cases. It was noted from the results that a lower expression of CD44 and CD24 was associated with an absence of nodal metastasis (Table 3).

**Table 3:** Details of nodal status and CD44 & CD24 expression OSCC, oral squamous cell carcinoma

		Sample size	CD 24+N+	CD24+N-	CD24-N+	CD24-N-	CD 44+N+	CD44+N-	CD44-N+	CD44-N-
1	Mirhashemi M et al. [11]	45	Not mentioned							
2	Tamatani T et al. [12]	70	9	32	5	24	Not mentioned	Not mentioned	Not mentioned	Not mentioned
3	Oliveira LR et al. [13]	157	Not mentioned							
4	Al-Magsoosi MJN et al. [14]	10	Not mentioned clearly							
5	Abdulmajeed AA et al. [15]	176	Not mentioned							
6	Saleem S et al. [16]	5	Not mentioned clearly							
7	Han J et al. [17]	6	Not mentioned clearly							
8	Todoroki K et al. [18]	50	2	9	5	34	6	17	1	26
9	de Moraes et al. [19]	36	Not mentioned specifically for OSCC							

CD44/CD24 Expression and Its Association to Disease-Free Survival and Overall Survival

Tamatani T et al. and Oliveira LR et al. evaluated the relation of CD44 or/and CD24 expression with disease-free survival and concluded that CD44 or CD24 or their co-expression was not associated with disease-free survival rate [12,13]. Olivera et al. and Todoroki K et al. also compared the CD44 and CD24 expression with overall survival rate and reported that double negatives (CD44-/CD24-) demonstrate a higher OS than other immunotypes (Table 2).

Quality Assessment of Included Articles

Assessment of the quality of the included studies was carried out using the Newcastle-Ottawa quality assessment scale. The details of cross-sectional studies are represented in Table 4, and the quality assessment of the case-control study is shown in Table 5.

**Table 4:** Summary of quality assessment of the cross-sectional studies included in the review

Quality assessment of the cross-sectional studies (Newcastle-Ottawa quality assessment scale)									
S..No	Authors and year of publication	Selection				Comparability	Outcome		Summary scores
		Representativeness of sample	Sample size	Ascertainment of the exposure	Non-respondents	The subject in different outcome groups are comparable based on the study design or analysis, confounding factors are controlled	Assessment of outcome	Statistical tests	
1	Mirhashemi M et al. [11]	*		*		*	**	*	6
2	Tamatani T [12]	*		**		*	*	*	6
3	Al-Magsoosi MJN [14]			*		*	*	*	4
4	Abdulmajeed AA [15]	*	*	**		*	**	*	8
5	Saleem S [16]			*		*	*	*	4
6	Todoroki K [18]	*		*		*	**	*	6
7	de Moraes et al. [19]	*		*		*	**	*	6

 

**Table 5:** Summary of quality assessment of the case-control study included in the review

Quality assessment of the case-control studies (Newcastle-Ottawa quality assessment scale)										
S.No	Authors and year of publication	Selection				Comparability	Exposure			Summary scores
		Adequate case definition	Representativeness of the cases	Selection of controls	Definition of controls	Comparability of cases and controls on the basis of the design and analysis	Ascertainment of exposure	Same method of ascertainment for cases and controls	Non-response rate	
1	Han J [17]	*	*		*	*		*		5

Summary scores were calculated and analyzed. The total scores ranged from four to eight for the cross-sectional studies, and the summary score was obtained as five for the case-control study.

Discussion

CSCs show a marked propensity for self-renewal and heterologous differentiation [4]. They play an important role in therapeutic resistance, metastasis, apoptotic resistance, and tumorigenesis [13,14,18]. CD44 and CD24 are engaged in different functions during cancer metastasis and progression. Despite the enormous amount of data available in the literature, their specificity and coexistence remain elusive in OSCC.

Most of the patients included in this review were males, and the tongue was the most common site of OSCC. It was also noted that both alcohol and tobacco use were frequently reported by OSCC patients. These agents are well known to increase the reactive oxygen species (ROS), cause DNA damage, and lead to carcinogenesis. Additionally, alcohol affects cancer stem properties, like differentiation and its maintenance, by increasing the production of ROS. CD44, a well-known cell adhesion glycoprotein, participates in tumor invasion and metastasis [8,12]. In this review, CD44 expression (65.73%) was found to be higher in OSCC compared to CD24. CD44v, an alternative splice variant of CD44, was also higher in OSCC patients (77.5%). CD44-positive stem cells have been identified in head and neck squamous cell carcinoma and various other cancers [17]. These cells participate in cellular functions including cell differentiation, tumor proliferation, production of chemokines, cytokines, and growth factors and is a receptor for hyaluronic acid (a component of ECM) [20]. CD44 is also known to act on specific receptors for hyaluronic acid and promote epithelial-mesenchymal transition.

It was noted that increased CD44 expression correlated with better differentiation. Similar results were reported by Koukourakis MI et al. in locally advanced head and neck cancers [21]. This could be attributed to the smaller number of MDSCC and PDSCC samples in the present review. As more than one subtype of CSCs may co-exist in the same tumor, there is also a possibility that CD44 positivity mainly characterizes the terminally differentiated tumor cells, which retain their specialized function. Other authors have hypothesized that the reduced expression of CD44 in poorly differentiated carcinomas may reflect loss of extracellular adhesion and increased invasive potential [22]. Clay MR et al. suggested that CD44+ cells are unlikely to be the pure population of CSCs, and further evaluation with a combination of other CSC markers is required [23].

CD24, on the other hand, modulates cell adhesion and other cellular functions through various signaling pathways, like MAPK, AKT/mTOR, EGFR, Src/STAT3, and miRNA-related pathways [24]. Concordant with the literature, the present review found that CD24 was expressed in patients with OSCC (61.18%). CD24 is known to promote tumor growth and metastasis in various malignancies, including OSCCs. This glycoprotein on the tumor cells interacts with P-selectin on endothelial cells and is implicated in malignant cell extravasation and binding to fibronectin in the extracellular matrix, promoting growth and metastasis. No difference in CD24 expression was observed between WDSCC, MDSCC, and PDSCC. This could be due to the smaller number of studies evaluating the expression of CD24 in different grades of OSCCs. Another difficulty lies in evaluating the different cut-off points and methodologies used in the included studies.

Recent research focuses on precisely identifying various stem cell markers, estimating their frequencies, and determining possible prognostic outcomes. An attempt was made to assess the diagnostic utility of CD44/CD24 combination in tumor development, metastasis, and overall survival of patients with OSCC. 33.81% of the cases were CD44-/CD24+, followed by CD44+CD24+ (25.6%), CD44-CD24- (23.67%), and CD44+CD24- (16.9%). Co-expression of both markers has been reported in various malignancies. Triple-negative breast carcinoma with CD44+/CD24- was linked to poor prognosis [25]. However, double-negative OSCC cases showed good clinical outcomes [13]. Todoroki K et al. suggested that CD44v3+/CD24- has anti-apoptotic effects [18]. In this review, predominantly, CD44-/CD24+ immunoexpression was reported in OSCC. Similar results were obtained by Ahmed MAH et al. in breast cancer patients and reported that CD44-/CD24+ expression is associated with high-grade tumors [26]. This suggests that more than one subpopulation of cells with stem cell properties may exist in the same cancer.

This systematic review also demonstrated that double negatives (CD44-/CD24-) have a higher overall survival rate than other immunotypes. Oliveira LR et al. reported that double-positive tumors (CD44+/CD24+) showed a 0% five-year overall survival rate [13]. CSCs give rise to more differentiated progenies, metastasize and initiate tumor growth in distant organs [27]. Grosse-Wilde A et al. reported that CD24+/CD44+ cells formed ten times more mammospheres when compared to CD44+/CD24- and CD24+/CD44- types [28]. The aggressiveness of such double-positive cells (CD44+/CD24+) was also reported by Goldman A et al. [29].

Epithelial-mesenchymal transition (EMT) is important for the tumor cells to escape anoikis, survive in circulation, and seed at a distant site. Mesenchymal epithelial transition (MET) is important for the carcinoma cells to form proliferative epithelial cells, leading to metastatic colonization [27]. CSCs are reported to have properties of EMT and MET [27]. These CSCs may accumulate mutations and adapt to the surroundings, further increasing the possibility of metastasis and decreasing overall survival. Circulating tumor cells (CTCs), both the mesenchymal-like CTCs and epithelial-like CTCs, have been reported in various carcinomas. When compared to individual CTCs, CTC clusters (possibly having both epithelial tumor cells and mesenchymal tumor cells) can generate a higher percentage of metastasis [30]. Polyclonal origin of metastasis and crucial cell-cell interactions have been identified in these CTC clusters. Grosse-Wilde A et al. evaluated the stemness of the hybrid epithelial-mesenchymal state of breast cancer cells and reported that CD24+/CD44- are more expressed in epithelial phenotype and CD24-/CD44+ in mesenchymal phenotypes [28]. Possibly, CD44+CD24+ cells may be seen in CTC clusters of OSCC with properties of both EMT and MET. This increases the chance of metastatic and local spread of the tumor, thereby decreasing the survival rate. 

The reason for the failure of most cancer treatments is the resistance of tumor cells to radiotherapy and chemotherapy. As the CTCs are able to self-renew and possess a high capability to repair DNA damage, they are resistant to various oncotherapies including chemotherapy and radiotherapy [18]. Resistance to chemotherapy and radiotherapy will result in treatment failure, reducing the survival rate of patients. This could be one of the reasons why double-positive CD44+/CD24+ shows a reduced survival rate in OSCC patients.

## Conclusions

In conclusion, both CD44 and CD24 were expressed in oral squamous cell carcinoma. CD44-/CD24+ immunoexpression was predominantly reported in OSCCs and double negatives (CD44-/CD24-) have a higher overall survival rate than other immunotypes. CD44/CD24 profile may be used on small incisional biopsies to predict the outcome and planning of treatment. Further studies are necessary to confirm this specific combination of CSCs and its relation to oral squamous cell carcinoma. This finding may help in developing new therapeutic targets to suppress cancer metastasis and provide a better long-term prognosis for these patients.
